# The Roles of H19 in Regulating Inflammation and Aging

**DOI:** 10.3389/fimmu.2020.579687

**Published:** 2020-10-26

**Authors:** Bin Wang, Chun Wai Suen, Haibin Ma, Yan Wang, Ling Kong, Dajiang Qin, Yuk Wai Wayne Lee, Gang Li

**Affiliations:** ^1^ The Chinese University of Hong Kong (CUHK)—Guangzhou Regenerative Medicine and Health Guangdong Laboratory (GDL), Advanced Institute for Regenerative MedicineBioland Laboratory (Guangzhou Regenerative Medicine and Health Guangdong Laboratory), Guangzhou, China; ^2^ Department of Orthopaedics & Traumatology, Stem Cells and Regenerative Medicine Laboratory, Li Ka Shing Institute of Health Sciences, The Chinese University of Hong Kong, Prince of Wales Hospital, Shatin, Hong Kong, China; ^3^ Ministry of Education Key Laboratory for Regenerative Medicine, School of Biomedical Sciences, The Chinese University of Hong Kong, Hong Kong, China; ^4^ Innovation Center for Translational Medicine, The Fifth Affiliated Hospital of Guangzhou Medical University, Guangzhou, China; ^5^ Department of Haematology, University of Cambridge, Cambridge, United Kingdom

**Keywords:** long non-coding RNA, H19, aging, inflammation, stress, signaling network

## Abstract

Accumulating evidence suggests that long non-coding RNA H19 correlates with several aging processes. However, the role of H19 in aging remains unclear. Many studies have elucidated a close connection between H19 and inflammatory genes. Chronic systemic inflammation is an established factor associated with various diseases during aging. Thus, H19 might participate in the development of age-related diseases by interplay with inflammation and therefore provide a protective function against age-related diseases. We investigated the inflammatory gene network of H19 to understand its regulatory mechanisms. H19 usually controls gene expression by acting as a microRNA sponge, or through mir-675, or by leading various protein complexes to genes at the chromosome level. The regulatory gene network has been intensively studied, whereas the biogenesis of H19 remains largely unknown. This literature review found that the epithelial-mesenchymal transition (EMT) and an imprinting gene network (IGN) might link H19 with inflammation. Evidence indicates that EMT and IGN are also tightly controlled by environmental stress. We propose that H19 is a stress-induced long non-coding RNA. Because environmental stress is a recognized age-related factor, inflammation and H19 might serve as a therapeutic axis to fight against age-related diseases.

## Introduction

Aging is characterized by multiple organ malfunctions, and is an inevitable biological process. Chronic degeneration is a significant pathological phenomenon in various age-related diseases. Inflammation is a part of the self-defense response to pathogens and its associated damage functions in self-clearance mechanisms. Inflammation is also associated with age-dependent tumor pathogenesis. Cancer incidence increases with age, and is maximal at > 65 years of age [1]. Continuous inflammatory responses lead to tumors by inducing cell overgrowth; therefore, cancer has been regarded as a wound that never heals. Inflammation also leads to the pathogenesis of some chronic diseases, such as diabetes, Alzheimer disease, chronic kidney disease and osteoarthritis (1–4). Emerging evidence suggests that inflammation also plays critical roles in tissue regeneration by activating cell migration and proliferation or clearing undesired cells. The interplay between inflammation and tissue regeneration is complex. The effect of inflammatory reactions on tissue regeneration can vary depending on pathological stage (5, 6). During tissue repair, inflammation is immediately initiated by recruiting essential cells and activating aging-related inflammatory factors such as tissue necrosis factor (TNF-α), soluble TNF receptor II (TNFRII), C-reactive protein, interleukin (IL)-6, IL-18, IL-15, and adiponectin (7). Nevertheless, the molecular mechanisms of inflammation during aging remains elusive.

Long non-coding RNA (lncRNA) participates in regulating diverse biological processes, and their roles in aging have recently been focused. During aging, lncRNA modulate telomere length, senescence, heterochromatin formation, proteostasis, stem cell differentiation, cell cycle regulation, and intracellular communication (8). H19 was first identified as an imprinting lncRNA; it is abundantly expressed at the fetal stage and repressed postpartum except in the skeletal muscle system (9), where it controls the imprinting of a cluster of conserved genes that contain H19 itself and insulin-like growth factor 2 (IGF2). Intensive cancer studies have regarded H19 as an oncogene, so it might serve as a therapeutic target in cancer treatment (10). Evidence also suggests that H19 is a potential target of antiaging therapy. This review summarizes the network of H19 that is involved in regulating inflammation and aging.

## H19 Involvement in Age-Related Diseases

### H19 in Diabetes

The H19 is highly expressed in the fetus stage and adult skeletal muscle. Both the levels of H19 in fetuses with maternal diabetes and skeletal muscle of type 2 diabetes(T2D) patients decreased strongly (11, 12). In T2D patients, the H19 reduces the glucose intake *via* mir-let-7, inhibiting the expression of its essential target genes like insulin receptor (insr) and lipoprotein lipase (lpl) in skeletal muscle. (11)The expression of H19 is reduced in fetuses with maternal diabetes in that they have more methylation at the imprint control region (ICR) of H19-Igf2 which would enhance IGF2 and decrease H19 expression (12). In other studies, the DNA methylation profile of H19 could pass from diabetic mothers to offspring (13), implying that H19 expression is glucose-dependent.

### H19 in Osteoarthritis and Rheumatoid Arthritis

A negative regulatory role of H19 in inflammation has been proposed (14). H19 reduces TGF-β mRNA expression *via* mir-675, the encoding microRNA of H19 (15). Pro-inflammatory TGF-β drives the immune response of mesenchymal stem cells through smad3 (16). Thus, H19 might directly participate in the development of osteoarthritis (OA) though the TGF-β pathway. On the other hand, H19 is significantly upregulated in patients with osteoarthritis, whereas, its expression is suppressed by TNF-α and IL-1β (14). Interleukin-1β and TNF-α downregulate the expression of col2A1 and H19, indicating that H19 plays a positive role in cartilage repair. Proliferative chondrocytes in growth plates have a mesenchymal-like phenotype and lack epithelial surface markers. Since proliferative chondrocytes represent an intermediate stage between resting and hypertrophic chondrocytes, this temporary change in the epithelial mesenchymal transformation (EMT) could lead to endochondral calcification (17). Hence, H19 might contribute to cartilage repair by regulating the EMT.

In rheumatoid arthritis (RA), Tie-1, the pro-inflammation factor, is upregulated. Knockdown of Tie-1 in endothelial cells induces the simultaneous upregulation of H19 and downregulation of Toll-like receptor 2 (TLR2), indicating a potential anti- role of H19. The polarized expression of H19 and TLR2 suggests a link between H19 and the Toll-like signaling pathway (18). Physical and physiological stress usually mobilizes the innate immune system to protect the body from infection (19). Since the Toll-like receptor is associated with the innate immune response, H19 might also mediate stress-induced inflammation (20). Besides, The H19 inhibitor represses the proliferation and induce apoptosis of synovial cells through Notch signaling pathway (21). Highly expression of H19 is detected in the synovial tissue in RA patients. MAPK, EKR1/2, and PI3K activity control the level of H19. Especially, H19 transfected synovial fibroblast shows a significant change in TIMP2 expression, which indicates that H19 participates in extracellular remodeling in RA (22).

In summary, the role of H19 in arthritis is inflammation-related. H19 may contribute inflammation development in arthritis through interacting with the canonical inflammation pathway.

### H19 in Cardiovascular Diseases

Cardiomyocyte hypertrophy is an age-related disease. The selective clearance of senescent cells from the mouse heart leads to a decrease in cardiac hypertrophy (23). An RNA-sequencing analysis showed that H19 is upregulated in hypertrophied cardiomyocytes. A cellular study found that H19 inhibits hypertrophy factor through mir-675 by targeting CaMKIIδ (24). Overexpressed H19 promotes mammalian target of rapamycin (mTOR) phosphorylation and further inhibits cardiomyocyte autophagy (25). Since mTOR activation subsequently promotes inflammation, H19 might inhibit cardiomyocyte hypertrophy through an inflammatory pathway.

H19 is abundant in the heart in adulthood. It correlates with coronary artery disease. In cardiac ischemic, H19 is significantly upregulated under hypoxia conditions resulting in inhibition of Col1a1 and cardiac fibrosis (26). Cardiac hypertrophy and fibrosis correlate with aging-associate inflammation. In a cardiovascular infraction, H19 plays a protective role in fighting against these two aging-associated cardiac dysregulations.

On the contrary, H19 is repressed in the brain after birth. In ischemic stroke, H19 promotes M1 microglial polarization, which results in neuroinflammation (27). In atherosclerosis, another subtype of stroke, H19 promoting ACP5 protein and increased the risk of ischemic stroke (28). In ischemic stroke, H19 seems to play an adverse role in neurogenesis by decreasing the expression of Notch1 by inactivating the transcriptional activity of p53 (29). It has been proved that H19 polymorphisms correlate with the susceptibility ischemic stroke (30).

Cardiac fraction and ischemic stroke are all lead to hypoxia condition. These two diseases induce high expression of H19, but the role of H19 seems opposite between the brain and heart.

### H19 in Cancer

Cancer and aging are interrelated because aging is thought to be either pro-tumorigenic by regulating inflammation or tumor-suppressive by inducing cellular senescence. A chronic inflammatory environment promotes tumorigenesis, which involves various inflammatory factors, such as IL-6 and TNF-α (31). H19 is regarded as an oncofetal lncRNA and its upregulation in various cancers has been speculated (32). For example, H19 sponges let7a/let7b then mediate oxidative stress-induced IL-6 expression in cholangiocarcinoma (33, 34). In breast cancer, upregulation of H19 sponges with miR-152 promoting the proliferation and invasion of cancer cells *via* upregulating of DNMT1 (35). Indeed, the downregulation of H19 suppresses the expression of P53 that is involved in cell cycle regulation (36). In gastric cancer, downregulation of H19 associates with p53 which to target BAX inhibiting cell growth and inducing cell apoptosis in the AGS cell line (37). In the early years, scientists have tried to establish a unified theoretical model to describe the function of H19 in every stage of cancer. In cancer initiation, H19 responds to stress conditions such as reduced P53 and hypoxia, then further activates tumorigenic and supports tumor cell survival. H19 coordinates EMT (epithelial mesenchymal transition) to promote tumor metastasis. H19 even supports MET (mesenchymal epithelial transition) to promote tumor proliferation and colonization either in primary or in the secondary site (38). P53 and EMT are actually the core member of cancer development. This model is easy to follow and understand. It only covers the general function of H19. However, H19 has been widely studied in cancer. It is hard to establish a sample model to describe H19 while considering various specific cancer types. A recent study has analyzed the role of H19 in 24-types of cancer. Base on Cancer Genome Atlas (TCGA) pan-cancer datasets, they specifically examined H19 in pan-cancers and confirm that H19 mediates transcription factors as a microRNA sponge, various new H19 mediated TFs have been discovered (39). However, until now, the whole picture of causality between H19 and cancer remains unclear The bioinformatics analysis may provide a feasible paradigm to understand the role of H19 from a more comprehensive angle. whether H19 controls the inflammation response in tumors remains ambiguous.

To date, no direct evidence has shown that higher levels of H19 expression delays aging. **Table 1** summarizes recent studies of H19 in age-related disease. H19 is notably upregulated in all the listed age-related diseases in which inflammation plays a crucial role except Type II diabetes. Abnormal H19 expression in various age-related diseases, indicating that H19 could serve as a therapeutic target for preventing age-related diseases.

**Table 1:** Expression of H19 in various diseases.

*Tissue*	*Disease*	*Pathological expression*	*Relevant Signaling*
*Muscle (* 11 *)*	Type II diabetes	Down	PI3K/AKT
*Cartilage (* 14 *)*	Osteoarthritis	Up	Sox9/col2A1
*Synovial Tissue (* 22 *)*	Rheumatoid arthritis	Up	MKK/JNK
*Umbilical cord blood (* 40 *)*	Obesity	Up	N/A
*Plasma (* 41 *)* *Heart (24)*	Gastric cancer Cardiomyocyte hypertrophy	Up Up	N/A CaMKIIδ

## Interplay Between H19 and Inflammation

Accumulating evidence indicates that H19 might be an age-associated long non-coding RNA. The relationship between H19 and inflammation is unclear. H19 regulates the gene network of canonical inflammatory pathways including those of NFκB, p38/MAPK/mTOR, toll-like receptor, and TNF-α. H19 is a positive regulator of inflammation and it can activate NF-κB by regulating the expression of thioredoxin (42). H19 also has proinflammatory effects in atherosclerosis by enhancing p38 expression, which is a central modulator of the MAPK and NF-κB pathways (43). H19 sponges let7a/let7b then mediate oxidative stress-induced IL-6 expression in cholangiocarcinoma (44).

H19 and mTOR mediate PI3K and stat3 signaling, whereas stat3 is believed to promote inflammation in several diseases (45). The overexpression of H19 promotes mTOR phosphorylation that will result in suppressing the activity of mTOR and inhibiting cardiomyocyte autophagy (25). Similar regulation is also evident in keloid scarring, where H19 promotes the development of fibroblasts *via* the suppression of mTOR (46), which is regarded as a core target in anti-aging research. Many studies have examined the suppression of mTOR signaling to mimic the signaling of rapamycin and the phosphatase and tensin homolog (PTEN) pathway. Inflammation and hyperactivated mTOR are often associated (47), and thus H19 could be a potential suppressor of aging-related inflammation by suppressing mTOR activity. Interactions between H19 and pathways of inflammation are controversial. H19 promotes inflammation through several pathways and inhibits inflammation by suppressing mTOR. However, we summarized the literature and highlighted two groups of genes that might contribute to mediating H19 and inflammation to help further understand how H19 regulates inflammation.

### H19 Mediates Inflammation by Controlling the EMT

#### Inflammation Induces EMT

The EMT is involved in a multitude of biological processes, including embryonic development, wound healing, fibrosis, and cancer metastasis. Several markers have been identified that indicate transitional stages (48). Inflammatory factors attract cells from adjacent tissues, including neutrophils, macrophages, fibroblasts, and stem cells during the early phase of wound healing. The EMT is prerequisite for these cells to undergo migration. Fibrosis is a complication of wound healing caused by the excessive accumulation of fibroblast-secreted extracellular matrix and it is a major cause of dysfunctional injured tissue. Inflammatory factors are the major players during the early phase of fibrosis (49). Bone morphogenetic protein receptor, type IA (BMPR1A) mediates TNF-α-induced EMT in the skin and primary keratinocytes, indicating that smad1/5/8 signaling is also involved in inflammation-induced EMT (50).

Although the etiology of fibrosis is complex and tissue-specific, the EMT frequently occurs during the chronic inflammation-induced fibrosis of various tissues (51). The EMT also contributes to the progression and metastasis of cancer cells, and is involved in suppressing cell senescence and in developing resistance to radiotherapy and chemotherapy (52, 53). The EMT marker gene, snail2, can be stabilized through nuclear factor kappa B (NFκB) activation revealing a possible role of EMT in inflammation-induced cell migration (54). The activity of AKT/GSK-3β is required to stabilize snail in the TNF-α-induced EMT and metastasis (55)in colorectal cancer. In summary, because EMT is a downstream event of chronic inflammation, understanding the role of H19 in inflammation and how it interacts with EMT is imperative.

#### H19 Interacts With EMT by Regulating MicroRNA or Directly Controlling Epigenetic Modification

Many studies have indicated a relationship between H19 and EMT. H19 acts in colorectal cancer as a microRNA sponge for MiR-138 and MiR-200, which then target zinc finger e-box binding homeobox 1 (ZEB1) and ZEB2 that are important mesenchymal cell markers in EMT (56). H19 promotes the migration of pancreatic cancer cells by sponging let7, then targeting the EMT inducer, high mobility group AT-hook 2 (HMGA2) (57). Treating gallbladder carcinoma with TGF-β1 and IL-6 induces the expression of H19, which in turn, downregulates the protein abundance of E-cadherin and subsequently promotes the EMT (58). H19 exerts an inhibitory role on E-cadherin by binding to EZH2 (59)in bladder cancer.

In contrast, H19 can reverse the EMT and the migration of hepatocellular carcinoma cells by promoting expression of the MiR-200 family (60). Inconsistencies among experimental models and conditions resulted in conflicting data about regulation between H19 and EMT. H19 might either induce or reverse the EMT independently of EMT inducers. The expression of slug activated by TGF-β contributes to the H19/EMT regulatory axis by inhibiting MiR-200 that prevents H19 activation. Because H19 is an EMT inducer only when no other signals intervene, H19 might not be the exclusive decisional factor in EMT but will function like other EMT enhancers (61).

H19 might have similar functions as an EMT in mediating the inflammatory response. H19 also has opposing functions in the regulation of inflammation. H19 overexpression usually leads to enhanced expression of the p38/MAPK/NFkB pathway while directly suppressing mTOR/STAT3 pathway. The role of H19 in inflammation depends on context. Like its role in EMT, the function of H19 might shift according to changes in extracellular signals. H19 might activate inflammatory processes during wound healing together with EMT activation but suppress inflammation through the mTOR pathway in chronic systemic inflammation.

### H19 Interacts With Inflammation Through an Imprinted Gene Network (IGN)

A discussion of the role of H19 in an imprinted gene network (IGN) requires the introduction of the important insulin-like growth factor 2 (IGF2) gene. A classical modulation model exists between IGF2 and H19 because they are located in a nascent manner and each affects the function of the other through epigenetic regulation.

#### Interaction Model Between IGF2 and H19

As an imprinting gene, H19 co-regulates with the fetal-specific IGF2 gene, and its expression depends on the methylation level on the flanking sequence of H19 (62).

*The Igf2/H19* DMR is conserved among mammals including rats, mice and humans (63). H19 and IGF2 are usually paired because they are located next to each other at the same locus. This locus regulates the imprinting network while expression from parents is unbalanced. In this locus, the IGF2 and DLK1 genes come from a paternal allele, while H19, IGF2R, and SNRPN are maternally inherited. Insulin-like growth factor 2 functions is a positive regulator of metabolism and embryo growth (64). Current understanding of H19 is limited to its function as a non-coding RNA due to the lack of an open reading frame at the genomic level (65). H19 could simultaneously function as a tumor suppressor and an oncogene (32, 33, 66–69). H19 expression is regulated through the DNA methylation of its promoter. The expression of H19 and IGF2 differentially depend on the methylation status of ICR or DMR that are located upstream of H19 (70). Two theoretical models of the interaction between IGF2 and H19 in the DMR have been proposed. One is the enhancer competition model in which an enhancer is proposed downstream of the H19 locus to interact with IGF2 and H19. On the paternal allele, DMR is hypomethylated to prevent binding of the insulator protein CCCTC-binding factor (CTCF) (70). On the maternal allele, CTCF-disrupted interaction between IGF2 and its enhancer would lead to increased H19 expression. The other is the boundary model in which the three-dimensional structure of chromosome has been applied to illustrate CTCF regulation. The CTCF directly binds to the distal enhancer, which blocks IGF2 expression and cohesin stabilizes the binding (71). The CTCF plays a leading role in the regulation of H19 expression regardless of the model. Affecting the function is CTCF on several loci or directly altering CTCF expression at transcriptional level is crucial to H19 regulation.

#### H19 Negatively Controls the IGN

The coordinated IGN controls embryonic growth and development. This network contains maternal and paternal alleles, as well as growth-promoting and inhibiting genes. Expression levels among IGN rarely exceed 35%, indicating that unknown feedback mechanisms or extremal signaling from another network might be involved in IGN-regulated growth control (72). Meanwhile, expression of the IGN is downregulated holistically during the postnatally delayed growth at the somatic cell stage (73).

The imprinting gene, H19, controls the IGN, and MBD interacts with H19 to form a ribonucleoprotein complex that recruits histone lysine methyltransferases that suppress gene expression. Through this interaction, H19 increases levels of H3K9me3 at the DMR of other imprinting genes, including Igf2, Mest (Peg1) and Slc38a4, and suppresses IGF2 through MiR-675 (74, 75). The IGN genes, Igf2, Dlk1, Cdkn1c, Dcn, Peg3, and Mest, are upregulated during muscle injury and they can also be reassembled on the IGN in H19 knockout mice (76). H19 is a beneficial regulatory factor that can mediate the injury process by suppressing IGN expression. The activation of IGN after muscle injury raises the question of the role played by IGN in tissue regeneration, since inflammation is the first phase of this process. The IGN might control inflammatory signaling at the cellular level and contribute to the signaling of cells that response to extracellular stress.

The intermediate role of IGN between aging and inflammation has not been discussed and reviewed, but the interplay between inflammation and IGN core genes has been studied. Paternally expressed gene 3 (Peg3) reportedly helps to activate NFκB by binding with TRAF2, which is the cascade factor required to mediate the TNF-α-induced inflammatory response (77). Oxidized low-density lipoprotein (ox-LDL) stimulates elevated IGF2 expression and mediates the activation of IL6 and NFκB (78). Oxidative stress-induced loss of imprinting (LOI) of IGF2 is mediated through NFκB activation, and the binding of NFκB with CTCF indicates that NFκB helps to block CTCF from the H19 DMR (79). The interaction between NFκB and IGF2 might also contribute to abnormal age-related inflammation. Indeed, IGF2 and mTOR could have positive feedback regarding their own functions. The key target of mTOR in myogenesis is IGF2 and mTOR would directly regulate IGF2 expression through suppressing mir-125b (80). Not only is IGF2 activated by mTOR at a muscle-enhancer *via* a kinase-independent pathway (80) but it is also involved in tumorigenesis by binding to the upstream receptor, IGF1R, in the PI3K/AKT/mTOR pathway (81). Decorin is a proinflammatory ECM protein and its binding network has been summarized; decorin reduces IL-10 levels *via* micro-21 and protocadherin alpha gene cluster (PCDA) 4 in cancer cells (82). The abnormal expression of decorin in tendon development and repair leads to compromised mechanical properties of tendons with age (83). Another key gene in the network is a cyclin-dependent-kinase inhibitor (CDKN1C; p57), which is enhanced in an LDL-induced chronic inflammatory model (84). Thus, IGN might positively regulate inflammation signaling *via* IGF2 signaling. As in the IGF2/H19 model, increased IGF2 expression usually leads to suppressed H19 expression. In contrast, the LOI of IGF2 increases age-associated IGF2 expression (79, 85). Age-related LOI at the DMR in a maternal chromosome would induce abnormally high IGF2 expression, which might be involved in the higher incidence of cancer among elderly populations (86). The expression CTCF and its binding at the DMR are decreased in age-related cancer and cellular senescence (79, 87, 88). This indicates that H19 expression might decline with advancing age. Even though the relationship between IGN and inflammation has not yet been comprehensively reviewed, several imprinting genes are important for the positive regulation of inflammatory gene expression. Thus, H19 could negatively regulate the expression of these genes and subsequently mediate the inflammatory process. Additionally, since imprinting genes participate in growth control and aging, H19 should play a pivotal role in age-related inflammation.

## Molecular Mechanisms Controlling H19 Expression

H19 could be a potential therapeutic target of anti-aging by controlling inflammation. How the H19 expression is controlled must be understood before it can be manipulated. The regulatory mechanisms of H19 are currently under investigation and review. Unlike coding genes, the H19 locus lacks a promoter region, indicating that a unique mechanism decides its expression level.

### CCCTC-Binding Factor (CTCF) Controls H19/IGF2 Locus

Among these binding genes, the polycomb repressive complex 2 subunit (SUZ12) reassures the relationship between PRC2 and H19 expression. Poly (ADP-ribose) polymerase-1 (Parp-1) is involved in transcriptional regulation by modulating the chromatin structure, and H19 is upregulated in Parp-/- ES cells (89). Among the genes introduced into the binding network, mothers against decapentaplegic homolog (SMAD) 2 and SMAD3 are the key downstream regulators of TGF-β signaling. The CTCF-smad complex might function in the organization of chromatin cross-talk and this complex could provide hints regarding smad3 and CTCF binding (90). Small ubiquitin-like modifier (SUMO)3 is included in the network and this indicates that sumoylation, an important post-translational modification, also contributes to changes in the location or binding partner(s) of CTCF. The sumoylation of CTCF occurs near the c-myc P2 promoter (91). Although no direct evidence shows that CTCF bis sumoylated the H19 DMR, hypoxic stress induces desumoylation on CTCF and H19 upregulation (92). In addition, H19 RNA is upregulated under hypoxic stress (93). Sumoylation might act as a positive H19 regulator under hypoxia. The presence of UBE2I and UBC9 indicates that sumoylation on CTCF might proceed through the recruitment of the ubiquitin conjugating enzyme UBE2I (94). The SIN3A/HDAC complex is recruited to NKX3.2, a cartilage repressor, and the binding of CTCF and SIN3A implies the participation of H19 in cartilage development (95). The interaction between RNA polymerase II (POLR2A) and CTCF might explain why H19 is upregulated when CTCF binds to the upstream DMR of H19. The key proteins YBX1 and TAF1 can mediate the RNA polymerase II complex (96).

In summary, chromatin remodulation, a key process in epigenetic regulation, might play a key role in H19 modulation. Sumoylation is another important epigenetic modification that has the potential to alter H19 expression. The involvement of CTCF in regulating the activities of H19 on DMR indicates that H19 participates in a broad spectrum of cellular events.

### H19 Is a Stress Induced lncRNA

**Figure 1:**
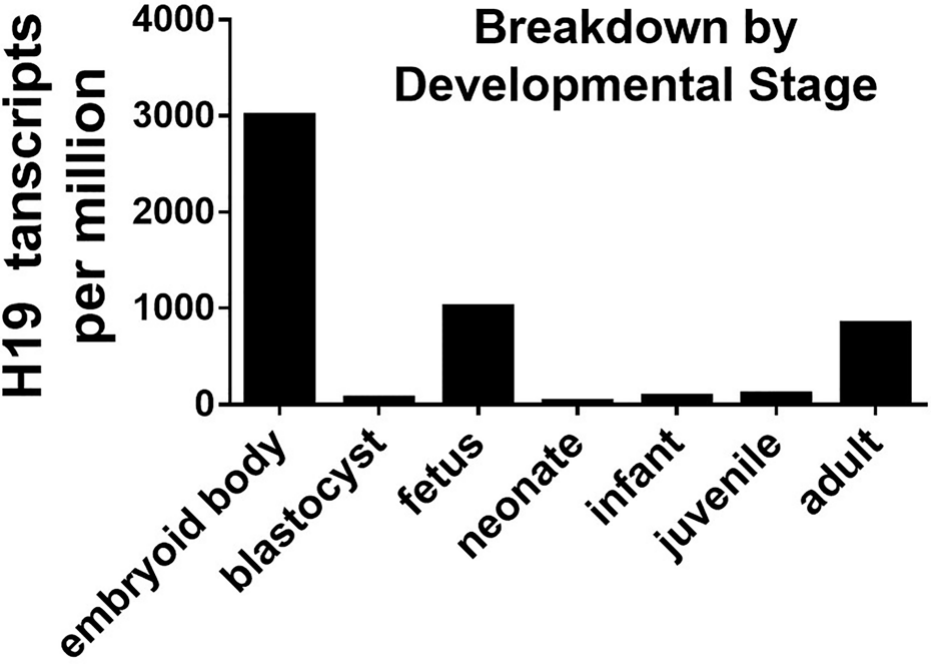
H19 EST profiile across developmental stage.

H19 and the inflammatory response are closely associated in the signaling network, yet the physiological role of H19 remains a matter of debate. H19 is apparently a stress-induced lncRNA, the expression of which changes in response to serum starvation and hypoxia stress-induced cell proliferation. These findings suggest that H19 mediates stress-induced tumor growth (97). However, whether the activation of H19 is detrimental or beneficial (97).to somatic cells is difficult to determine Stress-induced epigenetic regulation promotes H19 expression and audio stress on embryos results in the loss of methylation at the H19 locus, which leads to upregulated H19 expression (98). One study found relatively lower methylation levels on the H19 locus of epigenetic newborn twins derived from *in vitro* fertilization (IVF). Although IVF-induced stress has not been clearly defined, extracellular stress is sufficient to affect the epigenetic profile of H19 even after the long-term conception stage (99). Most study findings agree that H19 is an lncRNA that is expressed by the fetus and becomes suppressed after birth. The EST profile extracted from the NCBI database (https://www.ncbi.nlm.nih.gov/) (**Figure 1**) indicates that H19 expression does have another peak in adult humans. These data suggest that H19 becomes expressed and accumulated when adult humans are exposed to environmental stress (97).

The biological functions of the stress induced H19 gene in cellular responses remains unclear. However, considering the enhanced proliferation of stressed cells in serum, H19 might play a protective role. Acute inflammation helps tissue regeneration, while chronic inflammation might result in irreversible tissue injury. The expression of H19 is related to inflammation and corresponds to its function in tissue regeneration. The immediate post-injury elevation of H19 might help the migration and proliferation of cells at wound sites, and it is coordinated with inflammation at the early phase of regeneration. Since persistent activation of H19 might be a key factor in age-related tumorigenesis, H19 might similarly contribute to tumor growth and inflammation associated with aging.

### Epigenetic Regulation of H19

Since expression of the IGF2/H19 locus is based on the amount of DNA methylation at the DMR, the participation of DNA methyltransferases *de novo* or maintenance methylation are reasonable. Demethylases are also involved in regulating methylation, and several studies have shown that DNMT1 affects H19 expression in knockout mice and mouse models of overexpression (100, 101).

Either DNMT3a or DNMT3b knockout cells have hypermethylated H19 at the DMR, but not when DNMT3A/3B signals are knocked out (102). Besides, incubating cells with the methyltransferase inhibitor, 5-aza-2-deoxycytidine, reduces DMR methylation (103). The ten-eleven translocation (TET) family is also involved in the regulation of H19 methylation, because H19 demethylation in the ICR was removed when spermatogonia stem cells from Tet1 and Tet2 double knockout mice were reprogrammed (104).

Several important histone markers enriched around the H19 DMR during histone modification are associated with changes in H19 expression such as H3K9ac, H3K4me, H3K27me, and H3K9me (105–107). Levels of H3K9me2, which is the hallmark of silent chromatin, are increased around CTCF binding sites in mutant maternal alleles (108). The reduced H3K9me3 at the H19 promoter region promotes H19 expression in lung cancer cells (109). The overexpression of histone protein H3.1 linking to increased methylation at the DMR (110). These findings suggested that histone modification’s classical covenant model can be applied to H19 regulation at the chromatin level (111). Since repressive histone modification could be enriched around H19, the components of polycomb repressive complexes (PRC) might be linked to H19 expression. Maintenance of embryonic stem cells’ undifferentiated status requires the PRC 2 subunit (SUZ12), and SUZ12-deleted cells tend to have a global loss of H3K27me3 and significantly increased H19 expression (112). Another histone methyltransferase, SUV39h2, directly inhibits H19 expression in ovarian cancer (113). In addition, the acetylation of histones H3 and H4 affect the imprinting of the IGF2/h19 locus. This regulation is controlled by the chromatin condensation status, indicating that H3K9 methylation is also involved in H19 regulation (107). The expression of H19 depends on how the IGF2/H19 locus is modified according to the CTCF binding network, and several modifications might affect the level of H19 expression. These modifications have never been determined in age-related studies of H19 as a downstream target.

### H19 Controls Gene Expression Through Epigenetic Regulation

The non-coding RNA, H19, functions differently from coding genes. H19 might regulate inflammatory signaling as a microRNA precursor, a microRNA sponge or a chromatin structure modulator. H19 was originally identified as an endogenous RNA precursor of microRNA-675 during 2009 (114). H19 suppresses gene expression by overexpressing MiR-675 and inhibiting the post-transcriptional expression of target genes. H19 can block the TGF-β inhibitor, MiR-29b, during tenogenic differentiation (115). H19 facilitates the EMT by sponging MiR-138 and MiR-200a to promote ZEB1 and ZEB2 in colorectal cancer (56). These findings indicated that H19 functions as a competing endogenous RNA.

Apart from targeting microRNA, the H19 silencing gene can alter H3K9me3 or H3K27me3 histone modifications by recruiting the repressive chromatin modification complex. This salient chromatin structure suppresses the expression of nascent genes. H19 can interact with the PRC2 complex, such as by binding to the enhancer of Zeste 2 polycomb Repressive complex 2 subunits (EZH2) in cancer cells (59). H19 also binds methyl-CpG-binding domain protein 1 (MBD-1) in muscle cells when it does not form complexes with EZH2 or G9A (74). The difference in EZH2 binding indicates that the binding affinity of H19 and histone methyltransferase is tissue- or time-specific. The mechanism of the differential binding of H19 and EZH2 and the regulating factors involved in H19 functions in this binding have not yet been addressed. H19 might guide the recruitment of epigenetic modifiers to their DNA targets. How H19 chooses the target and whether the lncRNA has a distinct target profile remain elusive.

**Figure 2:**
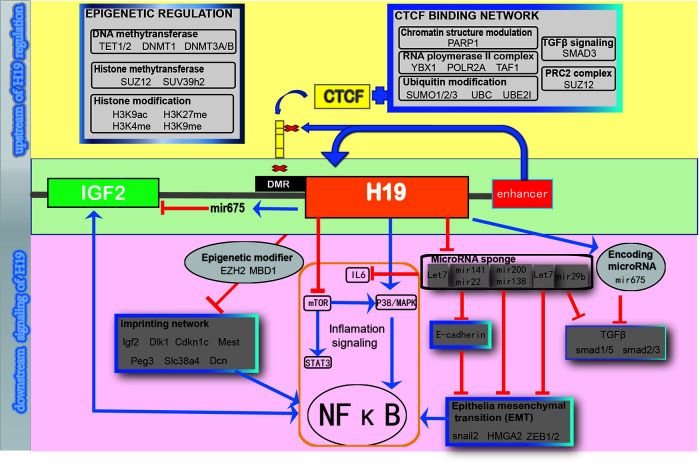
Schematic diagram of regulating H19 signaling network.

We concluded all the signaling networks mentioned in this review are a schematic diagram, which demonstrates the upstream and downstream signaling to summarize and to provide a comprehensive understanding of the regulation of H19 (**Figure 2**).

## Conclusion and Perspective

This review summarized the existing evidence of the expression trend of H19 during various age-related diseases. We discover a potential intermediate role of H19 correlated with the mentioned diseases. We then investigate the inflammatory gene network of H19 to understand its regulatory mechanisms. The literature review indicates epithelial-mesenchymal transition (EMT) and imprinting gene network (IGN) might link inflammation and H19. Our study also found that H19 is a stress-induced lncRNA. Because environmental stress is a recognized age-related factor, inflammation and H19 might serve as a therapeutic axis for age-related disease. The current review only focuses on the regulatory role of H19 in inflammation and aging. Due to H19 also control or participate in a broad spectrum of biological process. We proposed the following directions, in which H19 might also contribute to their functions.

### A Potential Therapeutic Strategy in Aging-Related Disease, Targeting H19 Controlled Cell Differentiation Through Inflammation Signaling

When we are getting old, body repair and tissue functions compromise due to the dysfunction of onsite stem/progenitor cells. Age-associated inflammation is the main reason for stem cell dysfunction. This linkage is mediated by NF-κB and another inflammation signaling (116, 117). H19 promotes the osteogenic differentiation of osteoblast cells through the MiR-675/smad3 pathway. Meanwhile, MiR-675 downregulates the expression of histone deacetylase (HDAC) 4/5, a smad3-recruited histone modifier that is involved in osteoblast differentiation (118). Through MiR-675, H19 directly inhibits HDAC4/5/6 expression in the adipogenic differentiation of bone marrow-derived mesenchymal stem cells. Meanwhile, forced expression of H19 also reduces the expression of CEBP-α and PPAR-γ that are important regulators of adipogenesis and inflammation (119). Obesity and metabolic dysfunction are thought to be associated with low-grade chronic inflammation and supporting evidence has been summarized (120). H19 might contribute to stem cell adipogenesis differentiation by regulating inflammatory cascades and then affecting the adipocyte metabolic functions. H19 might be considered as a potential anti-aging signaling target through the modulation of inflammation. Then further contribute to disease treatment through control cell differentiation.

### Role of H19 in Inherited Diseases, a Potential Direction to Understand the Relationship Between Inflammation, Aging, and Environmental Stimulus

Levels of H19 expression are usually high in inflammation-related diseases. H19 expression is abnormal in synovial tissues from patients with RA (22) and cartilage from those with OA patients (14). H19 is regarded as an inflammation-induced lncRNA that plays anabolic and protective roles in RA and OA, and DNA methylation at the H19 locus might be reduced by inflammatory stress (14). Due to the hereditary nature of RA and OA, long term systematically-induced energetic changes in H19 might contribute to the susceptibility of future generations to arthritis (72). Both RA and OA are age- and inflammation-related degenerative diseases. Investigating possible roles of H19 in these two diseases might provide further insight into how H19 regulates inflammation in response to environmental cues or even hereditary factors associated with aging.

## Author Contributions

BW: concept and design. BW and C-WS: manuscript writing. All authors contributed to the article and approved the submitted version. YL and GL: review of final manuscript.

## Funding

This work was partially supported by grants from the National Natural Science Foundation of China (81772322); the Hong Kong Government Research Grant Council, General Research Fund (14120118, 14160917, 9054014 N_CityU102/15, C7030-18G and T13-402/17-N); the Hong Kong Innovation Technology Commission Funds (PRP/050/19FX and ITS/448/18); and the Hong Kong Medical Research Funds (16170951 32 and 17180831). This study also received support from the SMART program, Lui Che Woo Institute of Innovative Medicine, The Chinese University of Hong Kong.

## Conflict of Interest

The authors declare that the research was conducted in the absence of any commercial or financial relationships that could be construed as a potential conflict of interest.
